# Non-Equilibrium Dynamics Contribute to Ion Selectivity in the KcsA Channel

**DOI:** 10.1371/journal.pone.0086079

**Published:** 2014-01-17

**Authors:** Van Ngo, Darko Stefanovski, Stephan Haas, Robert A. Farley

**Affiliations:** 1 Department of Physics and Astronomy, University of Southern California, Los Angeles, California, United States of America; 2 Department of Biomedical Sciences, Cedars Sinai Medical Center, Los Angeles, California, United States of America; 3 Department of Physiology and Biophysics, University of Southern California, Keck School of Medicine, Los Angeles, California, United States of America; University of Calgary, Canada

## Abstract

The ability of biological ion channels to conduct selected ions across cell membranes is critical for the survival of both animal and bacterial cells. Numerous investigations of ion selectivity have been conducted over more than 50 years, yet the mechanisms whereby the channels select certain ions and reject others are not well understood. Here we report a new application of Jarzynski’s Equality to investigate the mechanism of ion selectivity using non-equilibrium molecular dynamics simulations of Na^+^ and K^+^ ions moving through the KcsA channel. The simulations show that the selectivity filter of KcsA adapts and responds to the presence of the ions with structural rearrangements that are different for Na^+^ and K^+^. These structural rearrangements facilitate entry of K^+^ ions into the selectivity filter and permeation through the channel, and rejection of Na^+^ ions. A mechanistic model of ion selectivity by this channel based on the results of the simulations relates the structural rearrangement of the selectivity filter to the differential dehydration of ions and multiple-ion occupancy and describes a mechanism to efficiently select and conduct K^+^. Estimates of the K^+^/Na^+^ selectivity ratio and steady state ion conductance for KcsA from the simulations are in good quantitative agreement with experimental measurements. This model also accurately describes experimental observations of channel block by cytoplasmic Na^+^ ions, the “punch through” relief of channel block by cytoplasmic positive voltages, and is consistent with the knock-on mechanism of ion permeation.

## Introduction

Steady state concentration differences for inorganic ions between the cytoplasm and the extracellular bathing solution are essential for cell viability. Ion-selective channels embedded in the cell membranes that separate the cytoplasm from the extracellular solution facilitate the diffusion of specific ions across cell membranes and play important roles in cell physiology and pathophysiology. The ability of ion channels in neurons to discriminate between different monovalent cations, for example, underlies the initiation and propagation of action potentials, and the loss of ion selectivity in a G protein-gated K^+^-selective ion channel in mice leads to cell death and neurodegeneration [1]. The mechanism of ion selectivity by ion channels has been most intensively studied in K^+^-selective channels because of the availability of several atomic resolution protein structures of these channels. These structures show that the K^+^ ions are dehydrated when they are located within the selectivity filter of the channel, and it is likely that the process of dehydration of the ions is important to the mechanism of ion selectivity [2]. It was suggested by Bezanilla and Armstrong in 1972 that the mechanisms of ion selectivity would emphasize either selective binding of the ions in the selectivity filter of the channel or selective exclusion of ions from the selectivity filter [3]. Multiple binding sites for ions in the selectivity filter have also been suggested to be necessary for a high throughput flux of K^+^ [4]–[10], but how they are linked with the ion selectivity is still under debate [11]–[15].

In order to investigate the mechanism of ion dehydration and selectivity by potassium selective channels, numerous studies have employed free energy perturbation and umbrella sampling methods in molecular dynamics (MD) simulations [16]. MD simulations of ion selectivity in the bacterial KcsA K^+^-selective channel have identified numerous parameters of the ion-protein interaction such as ligand field strength, coordination geometry or number, or the surrounding protein matrix, that are important factors for determining ion selectivity [9], [12], [15], [17]–[21]. These methods used in MD simulations are powerful approaches that can provide insight into selectivity mechanisms from free-energy barriers; however, they usually employ constraints or algorithms to achieve fast sampling of rare trajectories that are not readily available in straightforward molecular dynamics simulations. To quantify the selectivity of K^+^ over Na^+^, the methods are often used to compute free energy differences between equilibrium states for different ions bound by the channel. These equilibrium states are themselves the consequences of ion selectivity, however, and may not contain information about the mechanisms that led to these specific bound states.

Several studies indicate that the structure of the selectivity filter of KcsA may depend on the ionic environment. Zhou et al. showed that the selectivity filter of KcsA crystallized in 200 mM K^+^ contains multiple K^+^ ions and is compatible with high-throughput ion permeation. The selectivity filter of KcsA crystallized in 3 mM K^+^ contains only a single K^+^ ion near the entrance to the selectivity filter and represents a non-conducting state [22]. Zhou et al. proposed that ion conduction occurs only when the selectivity filter snaps into a conformation similar to that observed in the protein at high K^+^ concentrations. Our simulation system was designed in part to determine the consequences of presenting Na^+^ and K^+^ ions to the selectivity filter under conditions where the selectivity filter is not filled with ions. Since the open probability of KcsA is very small (<0.03) [23], these simulations represent a physiologically realistic situation. Grottesi et al. also showed in simulation studies that the conformational flexibility of the selectivity filter leading to specific ion-bound states is not only essential for ion selectivity [24], but is also linked with the gating of potassium channels. Shrivastava et al. simulated the movement of both Na^+^ and K^+^ ions that were initially placed in the S1 and S3 sites in the selectivity filter after 2 ns of relaxation. They found that the ions relaxed to different positions and that the selectivity filter became distorted, with the distortion in the presence of Na^+^ being larger than that in K^+^ [25]. Finally, Nimigean and Miller experimentally demonstrated that cytoplasmic Na^+^ blocks the channel for observable times [26]. It is not known, however, how high concentrations of K^+^ trigger formation of the conducting structure of the KcsA selectivity filter, nor what structural features of the selectivity filter promote blockage by Na^+^ and conduction of K^+^. In addition, although a number of studies using free-energy perturbation MD methods concluded that site S2 in the selectivity filter of KcsA is the most selective binding site for K^+^ over Na^+^ [7], [8], [17], [18], [21], [27], [28], simulations by Kim and Allen [29] have shown that a thermodynamic preference for binding K^+^ over Na^+^ does not exist in multiple-ion configurations of the selectivity filter, and that ion selectivity is based on selective exclusion of Na^+^ from the selectivity filter rather than on selective binding of K^+^ to sites within the selectivity filter.

To address these issues, we use a newly developed approach to take into account transient interactions between the ions, water, and the channel. These out of equilibrium interactions underlie the conformation flexibility of the selectivity filter to induce ion-binding sites and must be included in a mechanistic description of ion selectivity [12]–[15], [30]. We have developed a method based on Jarzynski’s Equality [31] that uses step-wise pulling protocols to generate and exponentially weigh trajectories of single K^+^ and Na^+^ ions first entering the selectivity filter. These trajectories are then used to compute free-energy changes in both equilibrium and non-equilibrium dynamics [32], [33]. Because the system is relaxed at the end of each pulling step, this method provides information about transient processes between non-equilibrium and full equilibrium (i.e., quasi-equilibrium) states to extract movement trajectories of the ions and the fluctuations of other atoms involved in ion selectivity. It allows direct estimates of conductance while other methods such as steered molecular dynamics simulations [34], [35], adaptive biasing force [36], free-energy perturbation [37] and umbrella sampling methods [38] may require additional corrections [12] or dynamics [8] to obtain an estimate of maximum conductance. These simulations of single Na^+^ and K^+^ ions pulled through the KcsA selectivity filter show that the most stable locations for K^+^ and Na^+^ ions in the selectivity filter of KcsA are not the same and that entry of K^+^ into the selectivity filter is favored over Na^+^ by approximately 3.7 kcal/mol. The simulations also show that the selectivity filter of the KcsA channel undergoes structural rearrangements in response to the ions as they start entering the filter, and that these rearrangements are different for Na^+^ and K^+^ ions. The structural changes that are induced in the selectivity filter in response to the different ions favor entry and permeation of K^+^ ions and rejection of Na^+^ ions at the entrance of the selectivity filter, and identify the earliest step in the mechanism of ion selectivity by this channel. This is the first step for multiple-ion permeation requiring multiple-binding sites favorable for only potassium ions. The simulation data agree not only with a number of other simulations and experiments, but also suggest a way to resolve the inconsistency between the results from Kim and Allen and other simulation results. The simulation results add a dimension of non-equilibrium dynamics based on Jarzynski’s Equality and step-wise pulling protocols to previous studies of ion selectivity.

## Materials and Methods

Molecular dynamics simulations were done using NAMD [39], [40] to pull single K^+^ and Na^+^ ions step-wise from the center of mass (*z* = 0) of the simulation system along the z-axis toward the extracellular surface of the membrane. The simulation system consisted of the tetrameric KcsA channel (PDB accession number 1K4C) embedded in a POPC lipid bilayer with associated water molecules. The 1K4C crystal structure of KcsA was obtained at high K^+^ concentration and is compatible with ion conduction [22]. We prepared the two simulation systems with either 0.4 M KCl or NaCl, which have no ion in the selectivity filter, and contain 47332 atoms in total. The periodic boundary condition was applied to the x, y and z directions. The systems were first thermalized at 310°K for 1.5 ns to melt the lipid membrane and exclude water molecules from the hydrophobic region of the lipid membrane while the ion channel is kept fixed [41]. This lipid membrane is found sufficiently melted to bind to the ion channel, meaning that water molecules cannot enter the region between the channel and lipid molecules by thermal motion [http://www.ks.uiuc.edu/Training/Tutorials]. Then the channel is unconstrained, and an ion was placed at the center of mass of the system at z = 0 Å in vestibule and coupled with a soft harmonic potential (k = 0.6 kcal/mol/Å^2^≈1.0 k_B_T/Å^2^) having *λ = *0 Å as the center of the potential. The system was further thermalized for 0.5 ns with harmonic constraints (spring constant = 10 kcal/mol/Å^2^) on the lipid membrane in all directions. The simulations were done in the absence of an applied voltage difference across the membrane. Details of the simulation protocols to vary *λ* and to compute work distributions and free-energy profiles are given below. Charmm27 force fields [42]–[44] were used with the corrections to the Lenard-Jones interactions between potassium sodium ions, and carbonyl oxygen atoms, which were proposed by Noskov et al. [17]. Simulations were run on the High Performance Computing Center at USC and on Ranger at the Texas Advanced Computer Center, through the XSEDE portal.

### Step-Wise Pulling Protocol to Analyze and Control Non-Equilibrium Dynamics

#### Theory

**Figure 1:**
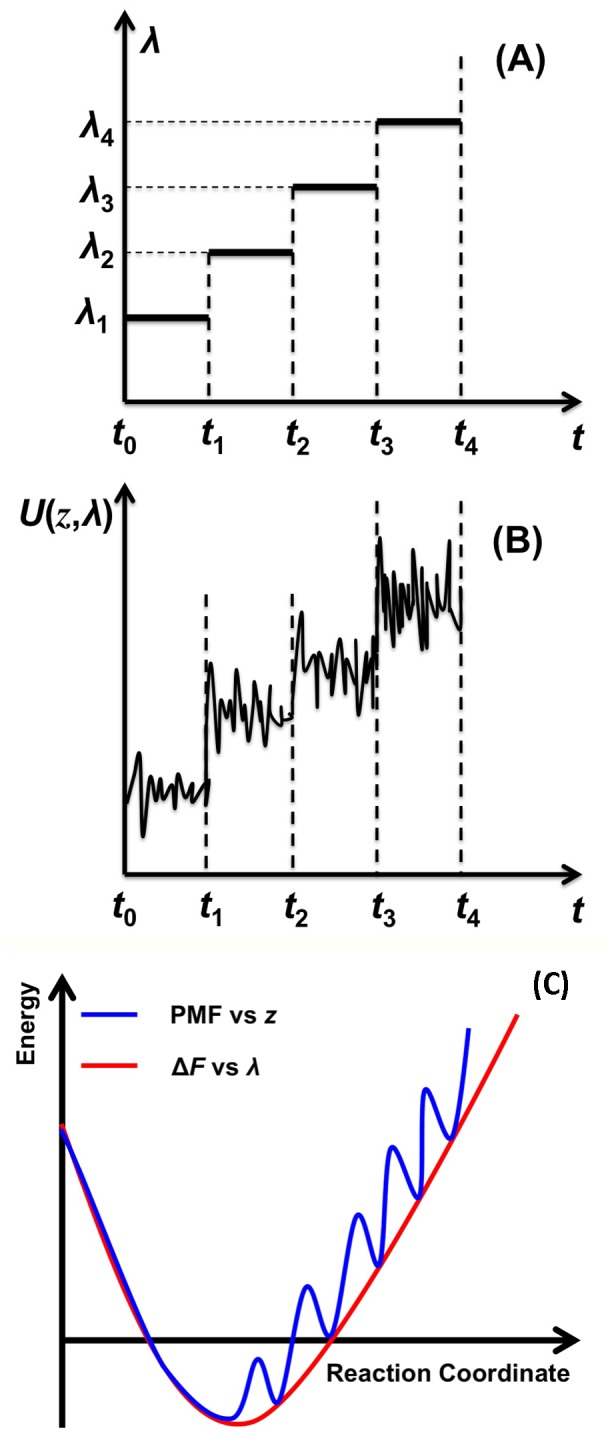
Schematic diagram for a step-wise pulling protocol. (A) Control parameter *λ*, and (B) expectation value of an applied potential versus time, U(z, *λ*) = k(z–*λ*)^2^/2. Coordinate z is the position of an ion along the z-direction. The pulling in the direction of increasing *λ* is called forward; the opposite direction of pulling is called backward. (C) Comparison between profiles for PMF (*blue*) and free energy changes (*red*) computed using our method.

The free energy profile is one of the most important features of ion channels that can be used to understand ion permeation. Recently, Ngo developed a computational technique based on the Jarzynski’s Equality, Eq. (1), and using step-wise pulling protocols that can be used to compute free energy profiles from work distributions in processes approaching equilibrium, without applying any additional algorithms for fast sampling of rare trajectories, such as are used in free energy perturbation and umbrella sampling methods. These step-wise pulling protocols [32], [33] require a finite number of pulling steps, and a relaxation time at each step (Figure 1A–B), which were determined by comparison with the potential of mean force computed by steered molecular dynamics simulations on stretching deca-alanine [34], [35]. The relaxation time is necessary to account for thermal effects [12], to allow the ion to move into stable or transient states, and to generate trajectories in work distributions. These trajectories are then exponentially weighted in the Jarzynski’s Equality. Importantly, this method is suitable to observe the transient processes from non-equilibrium to equilibrium, i.e., quasi-equilibrium.

**Figure 2:**
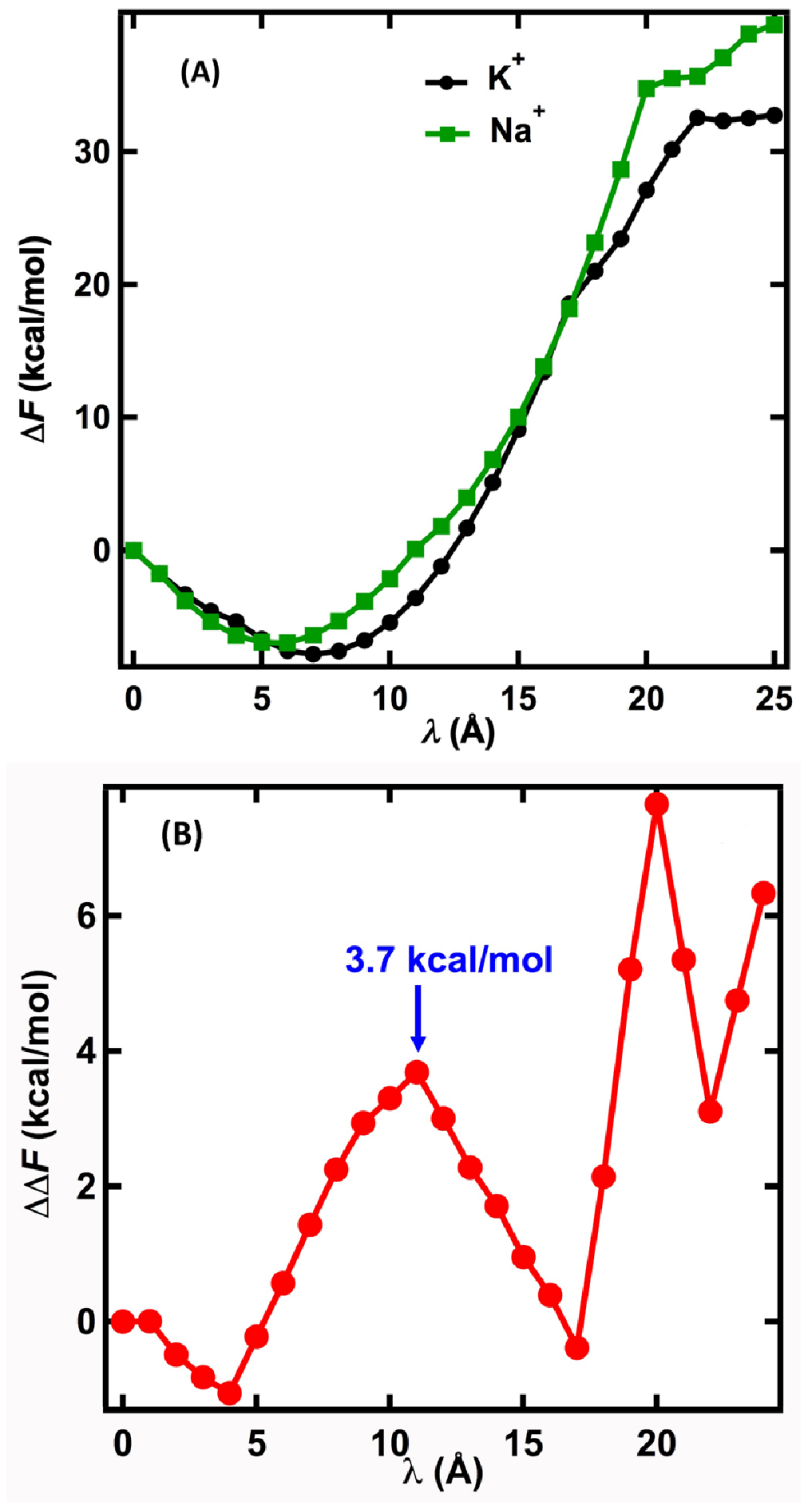
Free energy profiles for Na^+^ and K^+^ ions in the KcsA channel. (A) Free energy difference (ΔF) for Na^+^ and K^+^ ions after each pulling step (*λ*) compared to *λ* = 0. (B) Magnitude of the difference between Na^+^ and K^+^ ion free energy differences (ΔΔF) at each position of *λ* shown in (A). (C) The convergence of ΔΔF at *λ* = 11 Å during relaxation time τ. The uncertainty for computing free-energy profiles is about 1 kcal/mol.

A convergent free energy profile computed by the technique can be the same as the potential of mean force (PMF) when an applied harmonic potential is strong with large relaxation times and large number of sampling points of *λ*, which are also the essence of the thermodynamic integration [Eq. (2) or see Ref. [32] for more details]. However, when weak applied harmonic potentials are used, PMF and free energy profiles computed by the technique can be different (Figure 2C), but their free-energy differences between stable states determined by local minima of a PMF should be similar. The rationale is that weak applied harmonic potentials are insufficient to keep ions at transitional states (denoted by local maxima in the PMF). However, such a transition between two local minima should require the same amount of thermodynamic work, i.e., a free-energy change, regardless of applied potentials [32]. The advantage of using weak applied potentials in timescales of nanoseconds is that one can observe more subtle transition processes between the local minima than can be observed using strong potentials, which can easily bias the movements of ions. Furthermore, using strong potentials or other conventional techniques to compute PMF, it might not be possible to observe the dynamical adaptation and mutual responses of ions such as positions and velocities with respect to the changes of the selectivity filter.

The simulations using the step-wise pulling protocols generate the distributions of the *z*-coordinate *g_i_*(*z*) and averaged positions 〈*z*〉*_I_* of the ion. From *g_i_*(*z*) and 〈*z*〉*_i_*, free-energy changes can be computed as
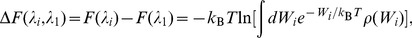
(1)

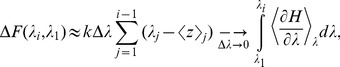
(2)

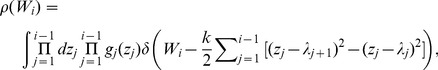
(3)where *λ_i_* = (*i*–1) Δ*λ* is the center of the applied potential, Δ*λ* is an increment, *H* is the Hamiltonian, *T* is temperature, *ρ*(*W_i_*) is the distribution function of work *W_i_* computed from *g_i_*(*z_j_*) with *z_j_* being all possible values of *z* at the *j*-th pulling step, and the Delta-Dirac function ∂ is used. Eqs. (1) and (2) hold for finite relaxation times indicating non-equilibrium, and infinite relaxation times indicating equilibrium, respectively. From the overlaps between successive *g_i_*(*z*), trajectories having small values of work *W* weighted by exp(–*ßW*) in Eq. (1) will have the largest contribution to the free energy profiles. We have found [33] that the most optimal reaction pathways having the largest contributions to free-energy profiles satisfy the principle of detailed-balance:
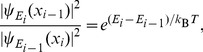
(4)where 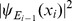 is a forward transition probability for the system at E_i–1_ moving into E_i_, and 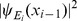 is a reverse transition probability for the system at Ei returning to E_i–1_. The solution of Eq. (4) can be represented by the Frank-Condon principle for general chemical reactions to occur, which requires certain overlaps among probability functions of reactants. Therefore, Equation (4) is useful to unveil the significant transition pathways occurring in quantum systems by computing the probability distributions of reactants and electrons.

#### Pulling protocols

To initiate forward pulling of one ion through the selectivity filter, the coupled system having *λ* = 0 Å was used as the initial state, and sequential pulling simulations were first performed along the z-axis in which the initial configurations for *λ_i_* were successively taken from the final configurations of *λ_i−_*_1_. An increment *λ_i_*–*λ_i_*_–1_ = Δ*λ* = 1.0 Å [32] yielding reliable results in stretching deca-alanine was used here. After each Δ*λ* step, a spatial displacement of the ion that depends on the interaction of the ion with the system as well as on the harmonic pulling potential may occur. Table 1 shows the resultant positions along the z-axis for both Na^+^ and K^+^ during step-wise pulling through the KcsA channel as described above. Note that for some positions of the ions, small incremental increases in pulling force associated with Δ*λ* do not result in movement of the ions. In each sequential step, the systems characterized by 25 values of *λ* from 0 to 24 Å are relaxed for *τ*_1_ = 0.5 ns, which is a minimum time found in Ref. [32]. Then, the 25 simulations were run in parallel for *τ*_2_ (∼10 ns) of further relaxation to ensure the convergence of physical quantities (Figure 2C). This relaxation time *τ*_2_ is also about the order for the selectivity filter to dehydrate, transfer and re-dehydrate K^+^ ions in experiments [6], thus it is reasonable to have all relevant data at each pulling step to sufficiently describe the ion movement. Moreover, the parallel feature is more computationally efficient to collect such data than the steered MD simulations without using step-wise pulling protocols [32].

**Table 1:** Z-Coordinates (Å) for Stable Positions for Na^+^ and K^+^ Ions After Each Pulling Step (Δλ = 1 Å).

λ	Stable z-positions(Å) for K^+^	Stable z-positions(Å) for Na^+^
0	2.1, 4.4	0.4, 2.3, 4.9
1	2, 4.5	2.4, 5
2	4.6	5.2
3	4.7	5.2
4	4.8, 7	5.3
5	5.2, 7.1	5.5
6	7.1	5.5
7	7.2	5.6
8	7.2	5.6, 5.8
9	7.3	5.7, 7
10	7.4	6.7
11	7.5	5.8, 6.8, 8.8
12	7.6	6.8, 9
13	7.7	7, 8.8
14	7.8	9
15	7.8, 10.3	9
16	7.9	9.3
17	7.9, 10.3, 13.9	9.1
18	8, 10.3, 14	9.2
19	10.7, 15.7	9.3
20	10.6, 14.5, 16, 17	9.4
21	10.6, 15.8, 17.7	9.6, 11.8, 21.9
22	17.5, 22.7	9.5, 10.5, 22.4
23	18, 22.7	9.5, 10.5, 11.3, 15.7, 22.5
24	18.1, 23, 24.5	9.7, 11.7, 15.8, 17.5, 22.1, 24

**Figure 3:**
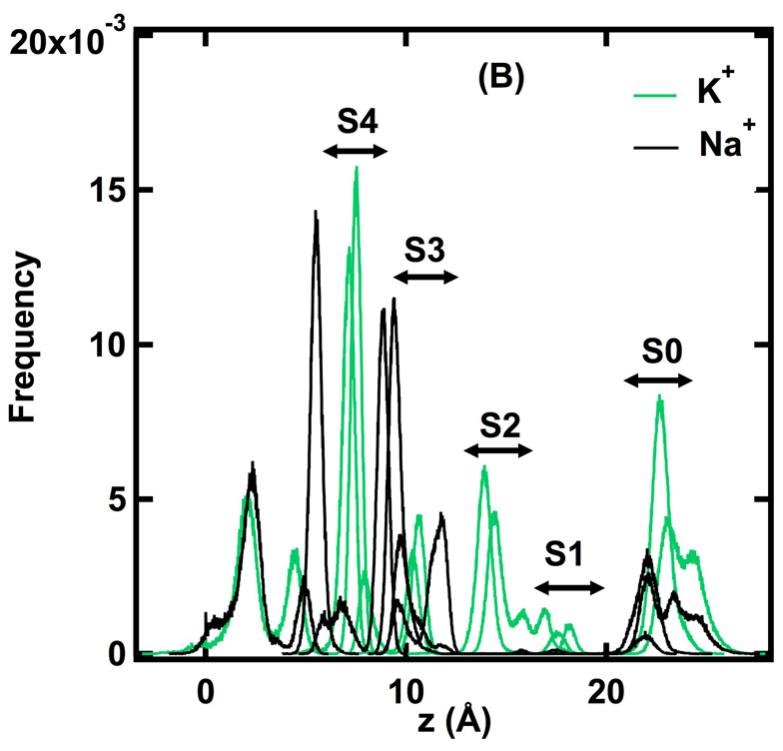
Histogram showing the frequency (probability) of finding the Na^+^ and K^+^ ions at each position (*z*) in the selectivity filter during step-wise pulling. The histogram is constructed from 25 pulling-step simulations for each ion. During relaxation of each pulling step, the positions of the pulled ions were collected every 100 fs and were put into bins of 0.01 Å width along the z-axis. Sites S0–S4 correspond to sites of stable K^+^ binding in the selectivity filter of KcsA. Stable Na^+^ positions are located in the plane of the carbonyl oxygen atoms that separate stable K^+^ binding sites. Note that Na^+^ ions are not stable within the selectivity filter at positions beyond the S2–S3 junction until they emerge from the channel at S0. Z = 0 corresponds to the center of mass of the protein-lipid-water system, within the central water-filled cavity in the transmembrane domain of the channel.

Na^+^ and K^+^ ions were pulled in increments (Δ*λ*) of 1 Å using a soft harmonic potential of 0.6 kcal/mol/Å^2^ that has its minimum value at *λ*. At the start of the pulling sequence *λ* is centered at the center of mass of the simulation system, z = 0. As *λ* is incremented by Δ*λ*, the spatial coordinates of the Na^+^ and K^+^ ions are unconstrained during the relaxation period τ. The z-coordinate of the ions at the end of *τ* is shown in the Table. Multiple values in the Table reflect the different z-coordinates observed in the simulations. A histogram of the frequency of occurrence of the different values for z for each ion at all values of *λ* is shown in Figure 3.

### Estimate of Channel Conductance from Work Distributions

Since work applied to an ionic charge *q* is equivalent to heat emitted by a resistance *R* = *G^–^* ^1^, we propose a simple expression of conductance
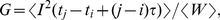
(5)where 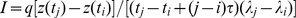, and *z*(*t_i_*) is the ion position along the axis of motion at time *t_i_*∈[0:*τ*] with *j*>*i* being the index of the pulling step. Here, the bracket denotes the average over any possible trajectories from the *i*-th to *j*-th pulling steps, and 〈*W*〉 is the averaged value of work for the trajectories. The physical interpretation of this current is that two electrodes are put at positions *λ_i_*<*λ_j_* to detect a charge *q* moving from *z*(*t_i_*) to *z*(*t_j_*). If there is a steady flow of a single ion moving with a constant velocity *v* measured within *τ* = *L/v*, then *I* = *qv*/*L* where *L* = *λ_j_*–*λ_i_* is the distance between the two applied electrodes and *t_j_*–*t_i_* = (*z*(*t_j_*)–*z*(*t_i_*))/*v*. Taking into account the fact that we sample *z*(*t_j_*), *t_j_*, *z*(*t_j_*) and *t_i_* independently (i.e., *t_j_*–*t_i_* can be negative), we collect their values such that *t_j_*–*t_i_*+(*j*–*i*)*τ* = (*z*(*t_j_*)–*z*(*t_i_*))/*v* to mimic a steady flow having *t_j_*–*t_i_*+(*j*–*i*)*τ*>0. In the picture of a steady flow, an electric field caused by the electrodes always performs one value of work *W* on the charge within a given duration, and *R* is related to a level of the friction or heat between the charge and filter, which is equal to *W*. In a non-equilibrium framework, the steady flow belongs to a set of many flows caused by various values of work mimicked by a harmonic applied potential. The heat of these flows is proportional to
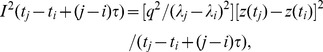
(6)whose average is equal to a diffusion coefficient Dij [45] multiplied with the factor in the square bracket. Therefore, for infinite relaxation times, one would obtain the convergent values of both numerator and denominator in Eq. (5), i.e., conductance G is a characteristic and convergent value. For a finite relaxation time, this conductance represents the averaged response of the selectivity filter toward all possible movements of an ion under non-equilibrium conditions. Note that G can be negative for a pair of pulling steps, which appears encounter-intuitive, but a total conductance is positive as described below.

Since work is more biased [32] for a pair having *j*>*i*+1 than a pair with *j* = *i*+1, we compute the conductance *G_i_*_+1_ for a pair of successive steps *i*-th and (*i*+1)-th, in which the work 〈*W_i+_*_1_〉 is averaged from *U*(*z*(*t_i_*),*λ_i_*+Δ*λ*)–*U*(*z*(*t_i_*),*λ_i_*) over all possible values of *z*(*t_i_*). Note that *G_i_*_+1_ can largely fluctuate along the selectivity filter due to the fact that 〈*W_i+_*_1_〉 and *D_ij_* are not the same for all pairs of successive steps. We compute a non-negative total conductance as follows
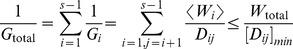
(7)


Since the total work W_total_ = ∑*_i_*〈*W_i_*〉 to enforce ions through the channel and the minimum of *D_ij_* are always positive, this total conductance is therefore positive. The first equality in Eq. (7) is known for a total resistance of multiple independent resistors in series. This suggests that the Eq. (7) is valid when all pairs of successive steps are uncorrelated.

## Results

### Free-energy Profiles

To simulate the movement of K^+^ or Na^+^ ions through the KcsA channel, a harmonic pulling potential that couples an ion's *z*-coordinate with the center of the potential (*λ*) was used. The protocol uses 24 discrete pulling steps with increments of 1.0 Å from *λ* = 0, and requires relaxation times τ at each step. The relaxation time permits collection of thermal distributions of the spatial coordinates that are used to identify stable positions of atoms, and to calculate work distributions that are used to compute free-energy profiles. Free energy profiles for the ions were calculated from the work distributions as described in Materials and Methods. In these simulations, each ion was initially hydrated at the center of mass of the simulation system, in the water-filled central cavity (vestibule) of KcsA, and was assigned a reference free energy value of zero. As the K^+^ and Na^+^ ions are pulled through the KcsA channel they encounter different free-energy barriers due to differences in the interactions between the ions and the channel. Although single Na^+^ or K^+^ ions were pulled through the channel in these simulations in order to simulate the interactions between the individual ions and the protein, the structure of the KcsA channel used was obtained at high K^+^ concentration, and represents the structure of the channel when the selectivity filter is occupied by multiple ions. Figure 2A shows the calculated value of the free energy difference (ΔF) for each ion as it moved from the center of mass of the system (z = 0) in response to sequential pulling steps (Δ*λ*) of 1 Å along the z-axis.

The free-energy profiles show that relative to the reference position, K^+^ has its free-energy minimum, −7.8 kcal/mol, when *λ* = 7 Å, and Na^+^ has its free-energy minimum, −7.0 kcal/mol, when *λ* = 5.5 Å. The large free-energy minima indicate that both ions are strongly attracted to these sites. Figure 2B–C shows that there is a peak in the convergent free-energy barrier difference (ΔΔF) of approximately 3.7 kcal/mol for the two ions at *λ* = 11 Å. The ΔΔF profile of the ions decreases at 12 Å<*λ*≤17 Å, and then increases. Note that a free-energy profile computed from this method has a direct physical meaning: since it is computed from distributions of work, it indicates an amount of thermodynamic energy, including any thermodynamic effects such as entropic effects, required to transform a system from one state to another state denoted by different values of *λ* (Figure 1). Thus, a difference in free-energy profiles for the two ions directly indicates an amount of work necessary for a transition between stable positions that are identified from a histogram of positions. Table 1 shows the corresponding z-coordinate values of the K^+^ and Na^+^ ions after each incremental pulling step Δλ, and histograms of these data showing the frequency of occurrence of each z-value throughout the step-wise pulling simulations is shown in Figure 3.

### Location of Stable Ion Binding Sites

The peaks in the histograms shown in Figure 3 identify stable positions of the K^+^ and Na^+^ ions in the KcsA channel during the 25 pulling simulations. The peaks in the histograms for K^+^ are in the same locations as the crystallographic K^+^ binding sites S4 (z = 7.1 Å), S3 (z = 10.4 Å), S2 (z = 13.6 Å), S1 (z = 17.0 Å), and also the S0 region (z = 23.1 Å) (22). Site S4 coincides with the location of K^+^ at the minimum of ΔF for K^+^ shown in Figure 2B (see also Table 1). The histograms for Na^+^ show that there is a very stable site for Na^+^ located in the vestibule just outside of the selectivity filter (z = 5.5 Å), coincident with the location of Na^+^ at the minimum in ΔF for Na^+^ (Figure 2A), and that within the selectivity filter Na^+^ is stabilized by the coordination of the planar arrangement of carbonyl oxygen atoms found between the K^+^ sites S4/S3 and S3/S2. The simulations indicate that there are no stable positions for Na^+^ within the selectivity filter beyond the S3/S2 junction. Using free energy perturbation MD simulations of ion binding in KcsA, Thompson et al. [46] and Kim and Allen [29] also found that the binding sites for Na^+^ and K^+^ in the selectivity filter of KcsA are different, and also suggested that selective permeation may involve barriers that exclude Na^+^ from the selectivity filter. The stable positions of the ions identified in the non-equilibrium simulations are the result of unconstrained motions of the ions in response to the incremental pulling potential. The peak in the ΔΔF profile at *λ* = 11 Å (Figure 2B) corresponds to the difference in the amount of work required to move a Na^+^ ion from its stable position in the vestibule (z≈5.5 Å) to the junction between S4 and S3 (z≈9.2 Å) compared to the amount of work needed to dislodge K^+^ from its stable position at S4. As is shown below, the movement of K^+^ into site S4 from the vestibule occurs with a high probability and is associated with little energy cost. The free energy difference of 3.7 kcal/mol derived from the non-equilibrium simulations indicates that the selectivity filter selects K^+^ ions over Na^+^ ions with a selectivity ratio for K^+^/Na^+^ of approximately 400. This value can be compared to the experimentally determined lower limit for K^+^/Na^+^ selectivity of 150 [47] and an upper estimate of ≈1,000 based on binding affinities [48], [49].

### Movement of Ions between Stable Binding Sites

**Figure 4:**
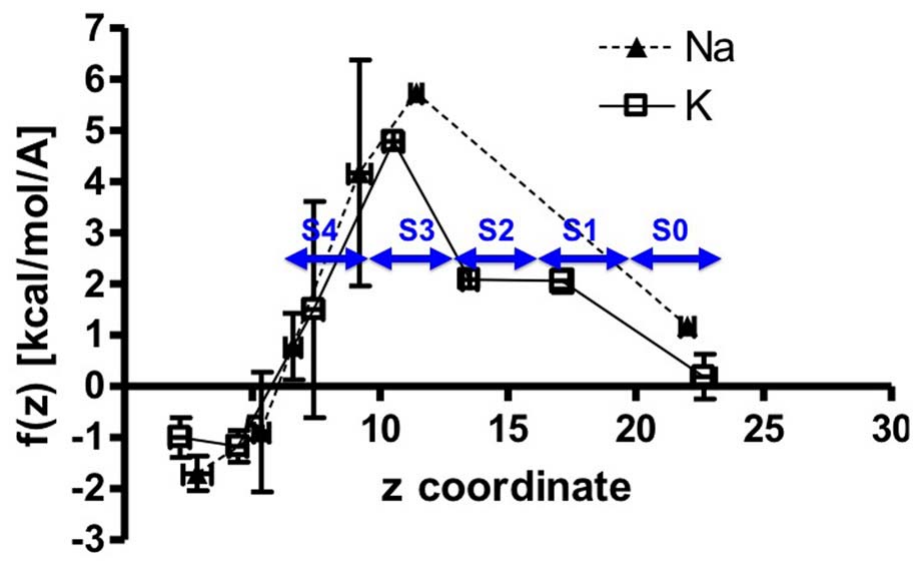
Force (kcal/mol/Å) on Na^+^ (solid *triangles*) or K^+^ (open *squares*) ions in the z-direction as they are pulled step-wise through the KcsA channel. Zero on the z-coordinate axis represents the center of mass of the system. Site S4 in the selectivity filter occurs approximately at z = 7.1 Å, site S3 approximately at z = 10.4 Å, site S2 approximately at z = 13.6 Å, and site S1 approximately at z = 17.0 Å. Histograms of the z positions of each of the two ions were created from all different values of *λ* (Figure 2). Univariate normal mixture decomposition implemented in STATA (STATA Corp., State College, TX) was used to determine the number of Gaussians that contribute to the complex distribution of each ion distribution in Figure 3. The mean of each Gaussian was associated with a stable position for the ions along the z-axis of the KcsA channel. The 95% confidence interval for each Gaussian was used to partition the force, f(z), to an equal number of segments of normal distributions. Means and standard deviations (SD) for the force were calculated for each segment. Mean values of f(z) are shown in the plot with SD estimates in both z- coordinate and f(z). We define the probability of transition from one stable position to another, P(z_i_−>z_i+1_), as the probability that the force in the current position, f(z_i_), is equal to or larger to the f(z_i+1_) in the subsequent position, P(f(z_i_)≥f(z_i+1_)). Thus, we assume that the transitions between to stable positions of the ions can occur by diffusion or by attraction to the subsequent position without any energy cost. For transitions between two stable points with small SD around the mean f(z), we consider that the probability of transition to the subsequent position is 1 if the mean f(z_i_)≥f(z_i+1_). Subsequently, the probability for the ion of going back once it reaches stable position z_i+1_ is 0. For transitions between stable position of the ion where either the mean for the force, f(z_i_), or the subsequent f(z_i+1_) has a small SD and the other one has a large SD, we calculate the probability P(f(z_i_)≥f(z_i+1_)) by focusing on the f(z) with the large SD. Within this given segment for f(z) we calculate the probability as sum of the observations that satisfy the inequality, f(z_i_)≥f(z_i+1_), divided by the total number of observations within that segment. Finally, for transitions between stable positions of the ion with coordinate z and a f(z) with very large SD, the probability of transition between two positions, P(f(z_i_)≥f(z_i+1_)), will be represented with P(f(z_i_)≥f(z_i+1_) ∩ f(z_i+1_)≤f(z_i_)) = P(f(z_i_)≥f(z_i+1_)) * P(f(z_i+1_)≤f(z_i_)). To calculate the two probabilities first find the observations in both segments that satisfy the inequality f(z_i_)≥f(z_i+1_) and then we divide the respective number by the total number of observations in the given segment. Furthermore, the above formula implies that we consider the two events independent.

The height of the peaks shown in Figure 3 does not represent relative equilibrium binding affinities at different positions within the channel for the ions but may be interpreted as probabilities for finding the ions at each value of z along the axis of the KcsA channel. These peaks were analyzed in more detail for the occurrence of each value of z at different values of *λ* and for the magnitude of the force associated with the ion at each value of z. The force on the ions in the z-direction at any value of z, f(z), is a result of the harmonic pulling force in the z-direction sampled at all values of *λ*. A histogram of frequency versus z-coordinate for each peak in Figure 3 at all values of *λ* showed that each peak is associated with a single distribution of z-values that can be approximated by a Gaussian distribution with a mean centered at the position of the maximum of the peak. Means and standard deviations (SD) were calculated for each peak. Histograms of f(z) associated with each peak in Figure 3, however, were sometimes approximated better by multiple Gaussian distributions with more than a single maximum value for the force. In these instances a Gaussian distribution was also used; however, a larger SD in f(z) resulted from this procedure than for peaks where a single Gaussian distribution was observed. Figure 4 shows a plot of the force f(z) associated with either Na^+^ or K^+^ ions at each z-coordinate position in the KcsA channel. Each data point corresponds to a different peak in Figure 3, with associated uncertainty in both z-position and force shown as SD.

**Figure 5:**
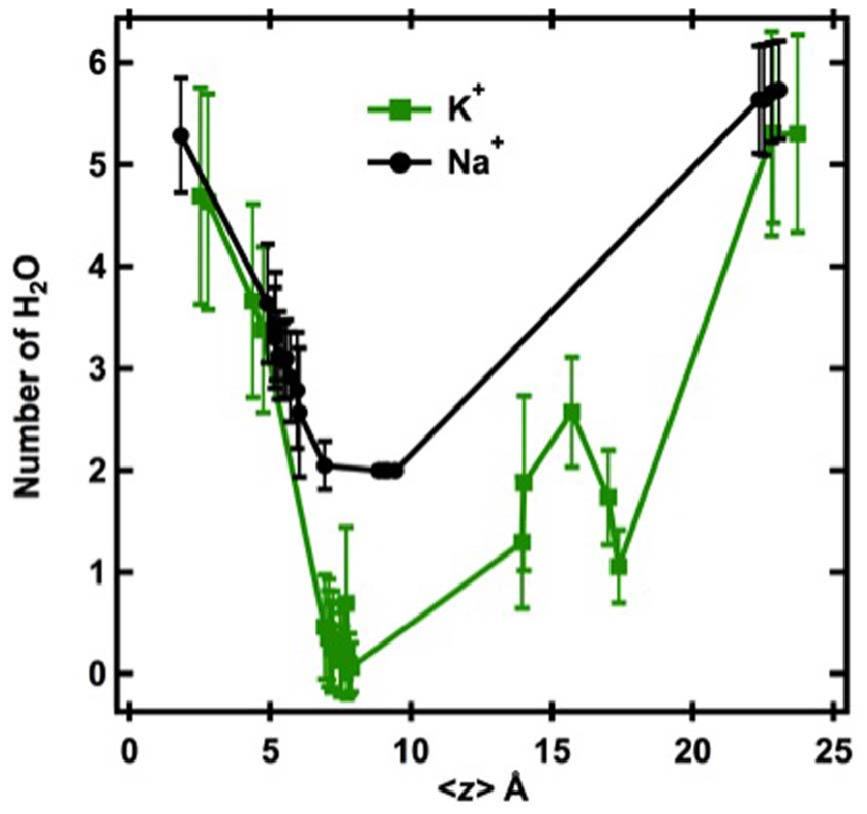
The number of water molecules within 3 Å of Na^+^ and K^+^ ions as they are pulled incrementally through the selectivity filter of KcsA from the cytoplasmic side of the membrane (*left*) to the extracellular surface of the membrane (*right*). On the x-axis, z = 0 corresponds to the center of mass of the system in the vestibule of the channel. The first data point on the left is a position in the vestibule below site S4. Site S4 is located at z≈7 Å. Values shown are means ± SD.

Figure 4 shows that the stable positions for the ions in the channel are defined fairly precisely, with SD of less than 1 Å. The force on the ions at each position is also fairly precisely determined when the ions are outside the selectivity filter in the vestibule of the channel (z<5 Å) or in the selectivity filter beyond the position of the carbonyl oxygen atoms that separate sites S4 and S3 (z >12 Å). The force on the ions in the region 5 Å<z<12 Å, however, is highly variable. This observation is significant because the ions are fully hydrated within the vestibule of the channel, whereas in site S4 the K^+^ ions are coordinated by an average of about 0.5 water molecules, and at all positions within the selectivity filter the Na^+^ ions are hydrated with at least two water molecules (Figure 5). It is likely, therefore, that the variation in force shown for the ions in the region 5 Å<z<12 Å is related to the mechanism of dehydration of the ions, which is the rate limiting step in ion permeation. For K^+^ ions there is a single stable position at S4 in the region 6.0 Å<z<8.5 Å and the SD for f(z) for this site is large. Within this region, 386 values of f(z) from a total of 1300 values were found to be <0, indicating that a K^+^ ion can jump spontaneously from the vestibule where it is hydrated to site S4 where it is dehydrated, with a probability of approximately 0.3. The probability that a K^+^ ion will jump spontaneously from site S4 to site S3 was calculated from the overlap of f(z) distributions at these two positions and is 0.11. These calculations reinforce the conclusion that S4 is a stable location for a single K^+^ ion within the selectivity filter and that K^+^ can enter the selectivity filter with little or no energy cost. For Na^+^ ions in the region 5 Å<z<12 Å there are two positions where the f(z) values are highly variable, corresponding to locations at the minimum of ΔF just outside the selectivity filter in the vestibule at z≈5.6 Å and in the selectivity filter between sites S4 and S3 at z≈9.1 Å. The probability of Na^+^ moving between these two positions was calculated from the overlap of the two f(z) histograms and was found to be 0.02. Thus, under the conditions of these simulations, Na^+^ is 15 times less likely to move from the vestibule to its stable position between S4 and S3 than K^+^ is to move from the vestibule into site S4. Alternatively, since rates of transitions between states can be considered to be probabilities per unit time, this result indicates that the rate of Na^+^ movement into the selectivity filter is at least 15 times less frequent than movement of K^+^ ions. Note that these simulation conditions are for single ions only and do not incorporate effects of multiple ions.

### Differential Dehydration of Na^+^ and K^+^ by the Selectivity Filter

Crystal structures of K^+^ ions in the selectivity filter of K^+^-selective channels indicate that the K^+^ ions are dehydrated, and the higher energy cost to dehydrate Na^+^ ions compared to K^+^ ions may be one of the key reasons that K^+^-selective channels exclude Na^+^ ions. Numerous physical parameters such as apparent hydration number, x-ray and neutron diffraction data, and the Jones-Dole viscosity B coefficient indicate that Na^+^ is strongly hydrated in aqueous solutions while K^+^ is weakly hydrated [50]. Figure 5 shows that the average number of water molecules within the first hydration shell (3 Å) of K^+^ and Na^+^ in the vestibule of KcsA (*z*<5 Å) is 5–6 for both ions, and that the number of bound water molecules fluctuates with different standard deviations for Na^+^ and K^+^ that reflect the shorter lifetime of water attached to K^+^ than to Na^+^ [51]. Some water accompanies K^+^ during entry into the selectivity filter, and the number of H_2_O molecules associated with K^+^ in S4 is approximately 0.5 on average. This is consistent with the ratio 0.9±0.2 of K^+^ to H_2_O in the multiple-ion conduction under electric fields as observed in much longer time MD simulations (∼µs) [10] than the single-ion pulling simulations. The figure also shows that Na^+^ is always accompanied by at least two water molecules when it moves through the selectivity filter. The absence of data points for Na^+^ in Figure 5 between site S3 and the extracellular solution is consistent with the histograms of the probability of finding the ions at specific positions within the selectivity filter (Figure 3), and indicates that there are no stable binding sites for Na^+^ ions between sites S3 and S0. Taking into account the average number of water molecules binding to the Na^+^, the effective diameter of Na^+^ is approximately 4.6–5.0 Å, which is significantly larger than the ionic diameter of K^+^. The dimensions of S4 in the KcsA crystal structure can easily accommodate a dehydrated K^+^ ion but not a hydrated Na^+^ ion.

### Structural Rearrangement of the Selectivity Filter in Response to Na^+^ and K^+^

**Figure 6:**
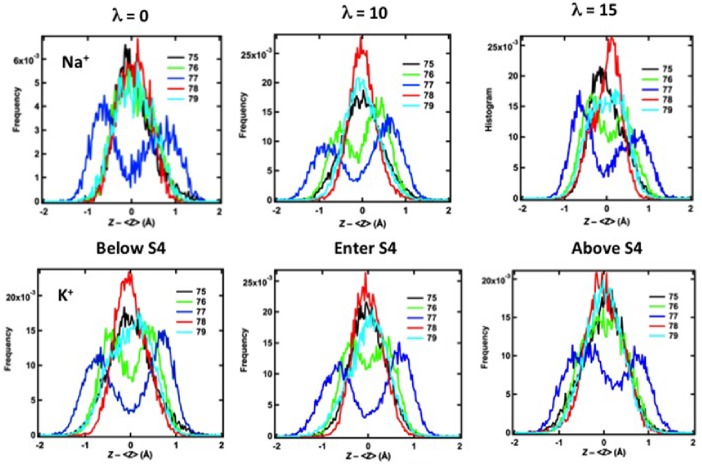
Positions of carbonyl oxygen atoms for amino acids T75–G79 in the KcsA selectivity filter during step-wise pulling of either Na^+^ (*top row*) or K^+^ (*bottom row*) from the vestibule toward the extracellular surface of the membrane. The y-axis is the frequency (probability) of finding the oxygen atoms at the position shown on the x-axis. Zero in the middle of the x-axis corresponds to the position of the atoms observed in the crystal structure. The carbonyl atoms corresponding to each amino acid in the tetrameric selectivity filter are shown in a single color, and each amino acid is represented by a different color (T75, *black*; V76, *green*; G77, *blue*; Y78, *red*; G79, *light blue*).

Rapid fluctuations in the positions of the carbonyl oxygen atoms of the amino acids in the KcsA selectivity filter were observed in the simulations. These fluctuations were examined in more details when either single Na^+^ or K^+^ was pulled from the vestibule into the selectivity filter since they directly quantify the flexibility of the selectivity filter. Allen et al. have suggested that the thermal fluctuations under external driving forces (from ions) are essential to the mechanism of ion selectivity, but these fluctuations have not been yet received sufficient attention [12]. Figure 6 shows that the positions of the carbonyl oxygen atoms depend on whether a Na^+^ ion (*upper panels*) or a K^+^ ion (*lower panels*) moves from the vestibule into the selectivity filter, altering the structure of the selectivity filter of KcsA in an ion-dependent manner. The figure shows histograms of the distribution of the positions of carbonyl oxygen atoms from the same amino acid in the four subunits of the tetrameric KcsA channel when the center of the harmonic pulling potential is located either just below S4 (*λ = *0), within S4 (*λ = *10), or just beyond S4 (*λ = *15). The histograms show results from the last 4 ns of each simulation.

In this figure, the value z = 0 corresponds to the position of the carbonyl oxygen atoms when they are arranged in a plane separating stable K^+^ binding sites, and deviations from this planar arrangement above and below the plane are shown to the left and right of z = 0. The effect of ion identity on the carbonyl oxygen atoms is observed most dramatically for the carbonyl oxygen atoms of V76 (*green traces*) that separate sites S3 and S2, although a similar pattern is seen for the carbonyl oxygen atoms of several of the other amino acids in the selectivity filter. When a Na^+^ ion is located in the vestibule below S4 (*λ = *0), the four carbonyl oxygen atoms of V76 are all found near the position observed for these atoms in the crystal structure, which defines a plane of oxygen atoms and a potential Na^+^ binding site between S3 and S2 [29], [46]. As the Na^+^ ion is pulled into S4 (*λ = *10) and beyond S4 (*λ = *15), the arrangement of the carbonyl oxygen atoms of V76 becomes disordered, disrupting the planar binding site for the Na^+^ ion between S3 and S2. Disruption of the planar binding site for Na^+^ between S3 and S2 will decrease the stability of Na^+^ binding to this site and hence reduce the probability that Na^+^ will move to this site. When K^+^ is located in the vestibule below S4, the carbonyl oxygen atoms of the four V76 residues are distributed between two positions located 0.5–1 Å above and below the plane. Movement of K^+^ into and beyond site S4 induces a planar arrangement of the carbonyl oxygen atoms of V76 and optimizes the structure of the S3 and S2 binding sites for the coordination of K^+^ by stabilizing the planar arrangement of carbonyl oxygen atoms that is seen in crystal structures. K^+^ entry into the selectivity filter also reduces the dispersion of the other carbonyl oxygen atoms in the selectivity filter around planar positions, creating an ordered structure in the selectivity filter that can accommodate multiple K^+^ ions.

This structural rearrangement of the selectivity filter induced by K^+^ represents a possible mechanism for the formation of the four contiguous binding sites for K^+^ in KcsA that Derebe et al. concluded is essential for K^+^ selectivity in this channel [15]. The distortion versus order caused by Na^+^ versus K^+^ is also consistent with the simulations of Shrivastava et al. [25], in which two Na^+^ ions were placed near S1 and S3, and cause the distortion during further relaxation of 2 ns. Our simulations show that such distortion begins even before Na^+^ ions enter the two sites.

### Estimation of Channel Conductance

**Figure 7:**
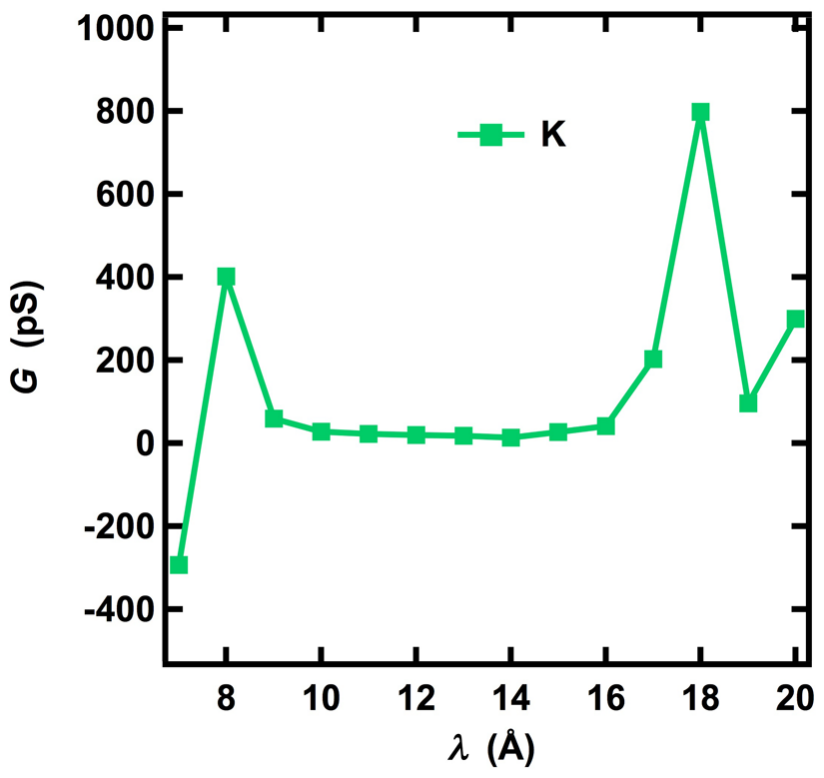
Calculated values of conductance (G) for a K^+^ ion pulled through the KcsA channel using a step-wise pulling protocol. Values of *λ* are the center of the harmonic pulling potential, and values between *λ* = 7 Å and *λ* = 17 Å occur when the center of the pulling potential is within the selectivity filter of KcsA. A negative value of G simply implies negative work (see Materials and Method) corresponding to the attraction (not resistance) from the selectivity filter at a pulling step. But for all pulling steps, the total conductance is always positive because the total work and diffusion coefficients are always positive.

Because the non-equilibrium simulations calculate work distributions for the movement of ions through the channel, an estimate of channel conductance can be obtained from the equivalence of work and the heat generated by an ionic current through a resistor. For a finite relaxation time, the calculated value of the conductance represents the averaged response of the selectivity filter to all possible movements of an ion in the non-equilibrium pulling processes at 0.4 M of salt concentration. We found that the total conductance values (*G*) associated with the pulling of single potassium and sodium ions are 2.9 and 1.8 pS at zero applied voltage, respectively. Even though the values of the total conductance are small, we found that the calculated average conductance *G*_ave_ of a single K^+^ ion in the region of the KcsA channel between sites S4 and S1 in the selectivity filter in the absence of a voltage gradient was 29 pS, and was higher outside of the selectivity filter (Figure 7). The measured value of the zero-voltage conductance of KcsA depends on KCl concentrations and is 97 pS at 0.1 M KCl and approximately 150 pS at 0.4 M KCl [47], values that are significantly higher than *G*_ave_. Both experimental data and kinetic models of ion occupancy in K^+^ channels indicate that the channel is occupied by 2–3 K^+^ ions during steady state conduction [4], [52]. If three K^+^ ions move independently through the selectivity filter within a unit of time, the value of conductance from our approach is estimated to be 29×3 = 87 pS, which is only half of the experimental value. Substituting the measured conductance of 150 pS into Eq. (5) for a steady ion flow with Δ*λ* = 1 Å and *z*(*t_j_*)–*z*(*t_j_*) = 0.5 Å [∼thermal fluctuations (12)] for *t_j_*–*t_i_*+(*j*–*i*)*τ = *10 ns [translocation time of K^+^ through the selectivity filter (6)], shows that the work done in this ion flow, 〈*W*〉≈0.6 kcal/mol∼*k*_B_ *T*, a value that is consistent with the “knock-on” mechanism of permeation in a barrier-free free-energy landscape for multiple ions [6]–[8], [53], [54]. Therefore, concerted movement of multiple ions incorporated into the diffusional and dissipative dynamical factors [8] that increase ion conductance in a multi-occupancy pore through the “knock-on” mechanism [4] is the likely reason for the deviation of the single conductance from experiments, in agreement with previous observations that ions do not move independently through the pore.

## Discussion

A mechanism for ion selectivity and K^+^ permeation by KcsA emerges from the results of these simulations. When a K^+^ ion approaches the selectivity filter from the vestibule, it is attracted to and stably bound to site S4 in the selectivity filter. Movement of K^+^ from the vestibule into S4 occurs spontaneously with a high probability and during this process the K^+^ ion is almost completely dehydrated. The dehydration processes of K^+^ and Na^+^ are significantly different in the simulations, in agreement with a number of experimental and simulation studies. Although the average number of water molecules (∼0.5) binding to K^+^ at S4 is slightly different from unity found in experimental experiments [6], [7] or 0.9±0.2 observed in simulations of K^+^ flow in Kv1.2 voltage-gated channels [10], this difference may be explained by lack of concerted K^+^ flow in our simulations. Elucidation of the mechanistic details of the dehydration process is likely to require quantum mechanical considerations, and the simulations performed here indicate that the organization of water molecules by the channel in the region just outside of the selectivity filter in the vestibule may be important for this mechanism (see below). Even in the absence of an electropositive driving force a K^+^ ion in site S4 will jump spontaneously from S4 to S3 with a modest probability. The structures of the S3 and S2 sites are themselves induced and stabilized by the presence of K^+^ in S4. Crystal structures show that a water molecule is found in the selectivity filter between adjacent K^+^ ions, and Markov chain models and calculations of the potential of mean force are consistent with the concerted movement of multiple K^+^ ions through the selectivity filter of KcsA, with sites S0 through S4 alternately occupied by a K^+^ ion or by a water molecule [8]. The simulations of single ion movement into the selectivity filter discussed here show that the region of the vestibule just outside of the selectivity filter is relatively depleted in water molecules when a K^+^ ion is located at its stable position in the vestibule or in site S4. The two or three water molecules in this region of the vestibule may be the source of the water molecules that separate adjacent K^+^ ions in the selectivity filter; however, in preliminary simulations of Na^+^ and K^+^ movement through a non-selective Na/K channel with a selectivity filter amino acid sequence similar to that of KcsA, occupancy of adjacent sites in the selectivity filter by K^+^ or Na^+^ ions is often observed (data not shown). Thus, these simulations suggest the possibility that water molecules may not always be interspersed between ions in the selectivity filter of KcsA. On the other hand, dewetting of the vestibule just outside the selectivity filter has been associated with hydrophobic collapse of the selectivity filter and loss of conduction during gating of K^+^ channels [10], and so the significance of the depletion of water in this region of the vestibule in KcsA ion selectivity is not entirely clear.

In contrast to the movement of K^+^, when a Na^+^ ion approaches the selectivity filter, it is stabilized in a location just outside the selectivity filter. Na^+^ must overcome an energy barrier approximately 3.7 kcal/mole higher than K^+^ to enter the selectivity filter, and the probability of a single Na^+^ ion moving spontaneously from the vestibule to the selectivity filter is only about 0.02 in the absence of a voltage gradient across the membrane. When a Na^+^ ion does enter the selectivity filter, it binds stably in the plane of carbonyl oxygen atoms between sites S4 and S3 together with approximately two water molecules. The movement of Na^+^ into this location induces disorder in the carbonyl oxygen atoms in the selectivity filter, however, and distorts potentially favorable binding sites for Na^+^ distal to this site. Thus, the Na^+^ ion is more likely to remain near the entrance to the selectivity filter than to move into the selectivity filter to the extracellular surface of the membrane unless it is driven by a strong electropositive potential (see below). A similar result was also observed in simulations by Shrivastava et al. [25], in which one Na^+^ prefers positions below the selectivity filter and two Na^+^ ions located near S1 and S3 distort the binding sites made of the carbonyl oxygen atoms during relaxation of 2 ns. Our data suggests that such distortion can be caused by a single Na^+^, and that Na^+^ is not able to bind to S1 for such a long time, hence explaining the “punch through” relief of cytoplasmic Na^+^ block as discussed below. Our data support the hypothesis originally made by Bezanilla and Armstrong [3] and more recently by Kim and Allen [29] that ion selectivity is based on selective exclusion from the selectivity filter rather than on selective binding to sites within the selectivity filter.

It is known that Na^+^ blocks the KcsA channel from an internal site suggested to be located within the vestibule of the channel [3], [26]. A stable location for Na^+^ in the vestibule was identified in these simulations (Figure 3) where the Na^+^ ion is stabilized by interactions with the hydroxyl groups of the four T75 residues of the channel. The simulations also indicate that Na^+^ can bind stably in the plane of the carbonyl oxygen atoms between S4 and S3, although under physiological conditions this site is not likely to be occupied. Current-voltage curves for KcsA show that block of the channel by cytoplasmic Na^+^ occurs at potentials near 100 mV and is relieved by internal positive potentials >200 mV. This effect of voltage on Na^+^ block has been called “punch through”. Na^+^ block and the concept of “punch through” can be understood in energetic and kinetic terms from the profile of f(z) for Na^+^ shown in Figure 4. The probability of a Na^+^ ion jumping from its stable position in the vestibule into the site between S4 and S3 is small (0.02). Furthermore, from the overlap of f(z) profiles for the Na^+^ site between S4 and S3 and the highest energy site for Na^+^ between S3 and S2, the probability of Na^+^ jumping to the latter site is calculated to be >0.5. Since there are no stable Na^+^ binding sites distal to this site in the selectivity filter, movement of Na^+^ occurs spontaneously from the site between S3 and S2 to the extracellular surface in the S0 region.

Our simulation data suggest that at membrane potentials <200 mV, Na^+^ would be stabilized in the site in the vestibule where ΔF is a minimum. At positive potentials >200 mV, Na^+^ ion would be dislodged from this site and would be driven into the site in the plane of carbonyl oxygen atoms between sites S4 and S3. The high probability of Na^+^ jumping from this location to the site between S3 and S2, however, even in the absence of a voltage gradient, and the absence of distal binding sites for Na^+^ in the selectivity filter, would favor movement of Na^+^ directly from the vestibule to the extracellular surface at high positive potentials. This mechanism is supported by our additional conclusion that work done during steady state ion conductance is approximately equal to *kT*, and by the conclusion of Berneche and Roux that the energy barriers to ion diffusion between adjacent sites in the selectivity filter are close to zero at moderately high positive values of the membrane potential [8]. The absence of overlap between histograms of f(z) for K^+^ at positions S1 and S0 indicates that backward movement of K^+^ from S0 to S1 occurs with a low probability, and this observation can explain the mild outward rectification observed for the KcsA channel [47].

The location of a stable site for Na^+^ in the vestibule just outside of the selectivity filter agrees with the simulations by Shrivastava et al. [25], and a K^+^ ion was observed near this location in crystal structures of KcsA and other K^+^ channels that were crystallized in the presence of potassium. Analysis of the reasons for the stability of Na^+^ at this location in the simulations provides some insight into the differential dehydration of Na^+^ and K^+^ as they enter the selectivity filter. Na^+^ is stabilized in the vestibule just outside the selectivity filter because the hydroxyl groups of four T75 residues at the entrance to the selectivity filter can substitute for four of the six water molecules around the hydrated Na^+^ ion, and because the remaining two water molecules are tightly bound to the Na^+^ ions. Because the motions of the hydroxyl groups are more restricted than the water molecules, this arrangement is energetically favored over fully hydrated Na^+^ in the vestibule. The hydroxyl oxygen atoms of T75 also replace four water molecules around the K^+^ ion, but in contrast to Na^+^, the weakly bound water molecules around the K^+^ ion are easily replaced by surrogate ligands. The restricted movement of the oxygen atoms on the T75 relative to the water oxygen atoms serves to further stabilize the K^+^ ion in site S4. Thus, movement of K^+^ into S4 occurs with no net energy cost for dehydration. This result suggests that further investigation into the role of T75 in ion selectivity in KcsA and in the non-selective NaK channel [30] by simulating Na^+^ and K^+^ movement into the selectivity filter of these channels containing amino acid substitutions at this position may provide valuable information about the difference in selectivity between these two channels.

The results reported here also suggest a way to resolve the discrepancy between simulations that support a selective binding mechanism versus those that favor a selective exclusion mechanism of ion selectivity. In simulations that conclude that the mechanism of ion selectivity is based on selective binding of the ions within the selectivity filter, free-energy differences for binding of the ions to each of the four sites S1–S4 in the crystal structure were calculated after replacement of K^+^ with Na^+^ in the same sites. In a recent simulation, however, using a similar replacement of K^+^ by Na^+^, it was suggested that there is no such special binding site for ion selectivity in multiple-ion configurations, and that ion selectivity is based on selective exclusion from the selectivity filter rather than on selective binding to sites within the selectivity filter [29]. The simulation data presented here indicate that there are no stable binding sites for Na^+^ near S2 or S1 (Figure 3) because the selectivity filter is already significantly distorted by the approach of Na^+^. This result does not exclude the possibility that the crystallographic S2 site is the most selective binding site for K^+^ over Na^+^, as previous simulations have concluded, however, the non-equilibrium simulations described here indicate that selectivity occurs even before Na^+^ enters S4, favoring the selective exclusion hypothesis.

One can also perform simulations of long duration (∼µs) [10] to mimic the conduction of K^+^ under electric fields, but this may not be possible for Na^+^ because the large selectivity ratio (∼400) of KcsA might require prohibitively long simulation times. Although permeation of only single K^+^ and Na^+^ ions was simulated using step-wise pulling protocols, the results of the simulations are consistent with other experimental and simulation data and mimic the realistic movements of ions in the channel. In these protocols, the weak harmonic potential and relaxation times allow both ions and the selectivity filter to respond mutually to each other. No assumptions about stable positions for the ions are assumed in these simulations, but rather the energetics of the system determine where the ions are stable in the channel. In this way, it was found that K^+^ easily enters its first stable position at S4 and induces the formation of the next binding sites, thus enabling subsequent K^+^ ions to follow with much less energy cost, as proposed in the “knock-on” mechanism of ion permeation [4], [27]. In contrast, Na^+^ prefers positions below the selectivity filter, but if it is forced into the selectivity filter, the selectivity filter responds by becoming more disordered, raising the energy cost for the entry of subsequent Na^+^ ions, i.e., dynamically rejecting Na^+^. This result not only agrees with the findings of Shrivastava et al. [25], but also suggests that if the distorted selectivity filter cannot accommodate multiple Na^+^ ions, then imposing multiple Na^+^ ions in simulations at the S1–S4 sites in the selectivity filter may not represent a physiologically realistic situation. An alternative approach may be to prepare a configuration of multiple Na^+^ ions by sequentially pulling two or three Na^+^ ions towards to the filter to observe how the ions and selectivity filter mutually respond to each other. It may then be possible to compare the energy differences between the pulling of multiple K^+^ and Na^+^ ions to determine whether they agree with the results by Kim and Allen [29]. These simulations are currently in progress in our laboratory.

In conclusion, simulations of non-equilibrium interactions between ions and the KcsA channel have identified an adaptation of the selectivity filter of the channel that adjusts the structure of the selectivity filter to favor entry of K^+^ ions into the selectivity filter with continued permeation of K^+^ through the membrane, and rejection of Na^+^ ions. This feature has been incorporated into a model for selective ion permeation in KcsA that is consistent with experimental measurements of conductance, rectification, and channel block. The mechanism of ion selectivity obtained from this analysis is able to extend results of previously published equilibrium MD simulations and to provide a new perspective on the mechanism of ion selectivity by KcsA. Although single Na^+^ or K^+^ ions were investigated in these simulations, the results obtained from stepwise pulling protocols combined with Jarzynski’s Equality are quantitatively consistent with several experimental measurements of selective ion permeation and indicate that this technique can also be applied to investigate the responses of the selectivity filter in the presence of multiple permeant ions. These results suggest a way to resolve current controversies about the mechanism of selective ion permeation. Although more subtle calculations based on quantum mechanics [19], [20], [55] can be used to obtain additional insight into the mechanism of the ion selectivity, our simulation data suggest that application of non-equilibrium molecular dynamics to ion channels provides an additional perspective on the dynamical adaptation and mutual responses of the ions and the channel.
